# The Response of Tissue Mast Cells to TLR3 Ligand Poly(I:C) Treatment

**DOI:** 10.1155/2020/2140694

**Published:** 2020-02-24

**Authors:** Piotr Witczak, Ewa Brzezińska-Błaszczyk, Justyna Agier

**Affiliations:** Department of Experimental Immunology, Faculty of Health Sciences, Medical University of Lodz, Lodz, Poland

## Abstract

Mast cells (MCs) are found mainly at the anatomical sites exposed to the external environment; thus, they are localized close to blood vessels, lymphatic vessels, and a multitude of immune cells. Moreover, those cells can recognize invading pathogens through a range of surface molecules known as pathogen recognition receptors (PRRs), mainly Toll-like receptors (TLRs). MCs are extensively engaged in the control and clearance of bacterial infections, but much less is known about their contribution to antiviral host response as well as pathomechanisms of virus-induced diseases. In the study, we employed *in vivo* differentiated mature tissue mast cells freshly isolated from rat peritoneal cavity. Here, we demonstrated that rat peritoneal mast cells (rPMCs) express viral dsRNA-specific TLR3 molecule (intracellularly and on the cell surface) as well as other proteins associated with cellular antiviral response: IRF3, type I and II IFN receptors, and MHC I. We found that exposure of rPMCs to viral dsRNA mimic, i.e., poly(I:C), induced transient upregulation of surface TLR3 (while temporarily decreased TLR3 intracellular expression), type II IFN receptor, and MHC I. TLR3 ligand-stimulated rPMCs did not degranulate but generated and/or released type I IFNs (IFN-*α* and IFN*β*) as well as proinflammatory lipid mediators (cysLTs), cytokines (TNF, IL-1*β*), and chemokines (CCL3, CXCL8). We documented that rPMC priming with poly(I:C) did not affect Fc*ε*RI-dependent degranulation. However, their costimulation with TLR3 agonist and anti-IgE led to a significant increase in cysLT and TNF secretion. Our findings confirm that MCs may serve as active participants in the antiviral immune response. Presented data on modulated Fc*ε*RI-mediated MC secretion of mediators upon poly(I:C) treatment suggests that dsRNA-type virus infection could influence the severity of allergic reactions.

## 1. Introduction

Mast cells (MCs) arise from CD34^+^ multipotent hematopoietic progenitor cells in the bone marrow. Released into peripheral blood, MC precursors are distributed to connective tissue throughout the body, where after tissue-specific differentiation, they mature and reside. Those cells have the capability of secreting *de novo* generated mediators and rapidly releasing preformed products stored in cytoplasmic granules [1–3]. MC extensive prevalence in almost every anatomical site, together with the secretion of a broad spectrum of mediators, enables those cells to be involved in various health- and/or disease-associated processes, ranging from homeostasis maintenance to inflammation and Fc*ε*RI-mediated allergic reactions [4–6].

An increasing body of evidence has now documented that MCs act as sentinel cells of innate immunity extensively engaged in the control and clearance of infections [7, 8]. Those cells are found mainly at the anatomical sites exposed to the external environment; thus, they are localized close to blood vessels, lymphatic vessels, and a multitude of immune cells [1–3]. Furthermore, MCs can “sense” and rapidly respond to invading pathogens through the range of surface and intracellular molecules known as pathogen recognition receptors (PRRs). Germline-encoded PRRs recognize unique bacterial, viral, fungal, or parasitic components known as pathogen-associated molecular patterns (PAMPs). The best-described receptors in MCs are Toll-like receptors (TLRs) activated by different pathogen- and damage-associated molecular patterns. So far, only a handful of studies indicate that MCs express C-type lectin receptors (CLRs) specialized in antifungal defense, NOD-like receptors (NLRs) detecting bacterial peptidoglycans, and RIG-like receptors (RLRs) relevant in viral sensing [9, 10].

MCs are well accepted as highly competent cells for host defense against bacteria. For instance, several microbial products have been shown to stimulate MCs through TLR2 and TLR4 to generate various proinflammatory humoral factors, including lipid mediators, cytokines, and chemokines [11–17]. Mainly, these cells exert several mechanisms of direct bacterial killing like the ability to phagocytose and intracellular digestion *via* oxidative and nonoxidative route [18] or, irrespective of phagocytosis, to form extracellular traps (MCETs), which can entrap and eliminate various bacterial materials [19]. Another noteworthy MC bactericidal activity is to generate and release antimicrobial peptides, such as defensins and cathelicidins [20, 21]. Moreover, there are reports of MCs presenting bacterial antigens through class I and II molecules of major histocompatibility complex (MHC) *in vitro* [22, 23] and *via* class I MHC *in vivo* [24], which support the concept of MC involvement in the promotion and development of adaptive immunity [25, 26].

Although the MC role in the host response to bacteria appears to be well-understood, their role in viral infections remains mostly unknown, without any detailed data. It might be speculated that those cells are capable of responding to virus-derived components and thereby involved in antiviral host defense on the one hand and pathomechanism of viral diseases on the other. According to some reports, MCs express PRRs responsible for the recognition of virus-derived PAMPs, mainly intracellular TLR3, TLR7, TLR9, and RIG-I [27–34]. The presence of the TLR3 transcript and protein is described and demonstrated both in MC lines and in various MCs differentiated *in vivo*. Therefore, TLR3 appears to be a crucial virus-sensing MC molecule that binds a wide range of viral antigens, i.e., double-stranded (ds)RNA, inducing an array of antiviral responses [35].

Since the functional activity of TLR3 in MCs is poorly understood, in this paper, we evaluated the effect of TLR3-specific synthetic mimic of viral dsRNA, i.e., polyinosinic-polycytidylic acid (poly(I:C)), on different aspects of native rat peritoneal MC (rPMC) biology: phenotype, degranulation, generation, and/or release of *de novo* synthesized mediators, cytokines, and chemokines. Considering MC's key role in allergic reactions [5, 6, 36], we also studied the effect of TLR3 agonist on the Fc*ε*RI-dependent rPMC response. Our results showed that rPMCs could respond to dsRNA analog by altering their phenotype, generating and/or secreting various proinflammatory and immunoregulatory humoral factors, and modulating Fc*ε*RI-mediated secretion.

## 2. Materials and Methods

### 2.1. Animals

The study was performed on female albino Wistar rats (Crl:WI; Charles River Laboratories) weighing ~250 g, aged three to four months. Standard storage conditions for animals were provided, i.e., room temperature (20 ± 2°C), artificial lighting for 12 h, and 12 h of darkness, in metal cages, with 5 rats in each. The animals were fed with LSM Murigran granulated fodder for rodents and water *ad libitum*. Isoflurane-induced anesthesia was carried out before animal decapitation. All efforts were made to minimize animal suffering.

### 2.2. Isolation of rPMCs

rPMCs were collected from peritoneal cavities by lavage with 50 mL of 1% Hank's balanced salt solution (HBSS) supplemented with 0.015% sodium bicarbonate (Life Technologies, Gaithersburg, MD, USA). After abdominal massage (90 sec), the cell suspension was obtained from peritoneal cavity, centrifuged (1200 rpm, 5 min, 20°C), and washed twice in complete Dulbecco's modified Eagle medium (cDMEM) containing DMEM supplemented with 10% fetal calf serum (FCS), 10 *μ*g/mL gentamicin, and 2 mM glutamine (1200 rpm, 5 min, 20°C) (Life Technologies). rPMCs were purified by centrifugation (1500 rpm, 15 min, 20°C) on isotonic 72.5% Percoll density gradient (Sigma-Aldrich, St. Louis, MO, USA). Subsequently, isolated rPMCs were centrifuged twice in cDMEM (150 g, 5 min, 20°C). After rinsing, rPMCs were counted and resuspended in an appropriate volume of cDMEM (for quantitative RT-PCR, flow cytometry analysis, western blotting, IFN-*α*, IFN-*β*, TNF, CCL3, and CXCL8 release measurements) or medium for rat rPMCs, containing 137 mM NaCl (Sigma-Aldrich), 2.7 mM KCl (Sigma-Aldrich), 1 mM MgCl_2_ (Sigma-Aldrich), 1 mM CaCl_2_ (Sigma-Aldrich), 10 mM HEPES (Sigma-Aldrich), 5.6 mM glucose (Sigma-Aldrich), and 1 mg/mL BSA (Sigma-Aldrich) (for histamine release assay and cysLT synthesis measurement), to obtain rPMC concentration of 1.5 × 10^6^ cells/mL. To acquire appropriate rPMC density and the number of samples *in the given* type of experiment, the proper number of animals was used. rPMCs were prepared with purity > 98%, as determined by metachromatic staining with toluidine blue (Sigma-Aldrich). The viability of rPMCs was over 98%, as determined by trypan blue (Sigma-Aldrich) exclusion assay. The results of the treated samples were compared to the control from a given experiment.

### 2.3. Western Blotting

For the determination of constitutive expression of TLR3, IRF3, IFNAR1, IFNGR1, and MHC I, immunoblotting was used. Purified rPMCs were lysed in ice-cold RIPA buffer (150 mM NaCl, 0.1% sodium dodecyl sulfate (SDS), 50 mM Tris-HCl, pH 8.0, with 1% Igepal CA-630 (NP-40), and 0.5% sodium deoxycholate) (Sigma-Aldrich) containing protease inhibitor cocktail (1 mM 4-(2-aminoethyl)benzenesulfonyl fluoride hydrochloride (AEBSF), 800 nM aprotinin, 50 *μ*M bestatin, 15 *μ*M E64, 10 *μ*M pepstatin A, 5 mM ethylenediaminetetraacetic acid (EDTA), and 20 *μ*M leupeptin) (Thermo Fisher Scientific, Rockford, IL, USA). Cells were incubated with lysis buffer on ice for 30 min, and undissolved residues were removed. Subsequently, the Bradford assay (Bio-Rad Laboratories, Inc., CA, USA) was used to analyze protein concentration in the lysates. The cell lysates (50 *μ*g of protein) were separated on NuPAGE 10% Bis-Tris Gel (Life Technologies) and then transferred to polyvinylidene difluoride (PVDF) membrane. Protein expression was detected using rabbit anti-TLR3 (1 : 500 dilution), anti-interferon regulatory factor (IRF)3 (1 : 500) (LifeSpan BioSciences, Inc., Seattle, WA, USA), anti-interferon-*α* receptor (IFNAR)1 (1 : 1000), anti-interferon-*γ* receptor (IFNGR)1 (1 : 500) (Novus Biologicals, Littleton, CO, USA), and anti-MHC I (1 : 1000) (Abgent, San Diego, CA, USA) antibodies. Horseradish peroxidase- (HRP-) conjugated goat anti-rabbit IgG (1 : 300 dilution) (Bioss Inc., Woburn, MA, USA) were applied as secondary antibodies. All proteins were visualized by enhanced chemiluminescence (ECL) system using the ECL chemiluminescent substrate reagent kit (Life Technologies) according to the manufacturer's protocol. Equivalent protein loading per lane was confirmed by stripping and immunoblotting the membranes with a rabbit anti-*β*-actin antibody (Abnova Corporation, Taipei, Taiwan). The developed images were scanned, and the protein band intensity was quantified by ImageJ software.

### 2.4. Flow Cytometry

Constitutive TLR3, IRF3, IFNAR1, IFNGR1, and MHC I expressions were determined using flow cytometry. Purified rPMCs at a concentration of 10^6^ cells/mL were fixed overnight with CellFIX solution (BD Biosciences, San Jose, CA, USA) at 4°C, then rinsed twice in 1x PBS with 0.05% Tween 20 (PBS-Tween) (Sigma-Aldrich) and resuspended in 1x PBS. After fixation, the cells were incubated with rabbit anti-TLR3 at a final concentration of 5 *μ*g/10^6^ cells, anti-IRF3 (2.5 *μ*g/10^6^ cells), anti-IFNAR1 (5 *μ*g/10^6^ cells), anti-IFNGR1 (2.5 *μ*g/10^6^ cells), and anti-MHC I (5 *μ*g/10^6^ cells) antibodies at 4°C for 1 h. Next, after dual rinsing with PBS-Tween, the cells were stained with FITC-labelled goat anti-rabbit IgG antibodies (Bioss Inc.) at a final concentration of 5 *μ*g/mL at 4°C for 1 h in the dark. Following incubation, cells were washed twice with PBS-Tween and resuspended in 100 *μ*L 1x PBS before the fluorescence measurements. For control, rPMCs were stained with rabbit IgG isotype control (R&D Systems, Minneapolis, USA) with irrelevant specificity. The primary antibody was not added to the sample to certify the nonspecific binding of the secondary antibody. For the analysis of intracellular protein expression, under the same experimental conditions, the fixed cells were permeabilized with 0.01% saponin (Sigma-Aldrich) in 1x PBS for 5 min at room temperature before staining with primary and secondary antibodies diluted in 0.01% saponin in 1x PBS. For the assessment of induced expression, the purified rPMCs were incubated with medium alone (constitutive expression) or poly(I:C) (Invivogen, San Diego, CA, USA) at a final concentration of 10 *μ*g/mL for 6 or 12 h at 37°C in a humidified atmosphere with 5% CO_2_. At least ten thousand events were acquired per sample and analyzed using FACS Canto II flow cytofluorimeter with FACSDiva software (BD Biosciences). Results were demonstrated as a percentage of mean fluorescence intensity (MFI) of unstimulated cells (taken as 100%). After each period of incubation, mast cell viability was assessed using a trypan blue (Sigma-Aldrich) exclusion test.

### 2.5. Quantitative RT-PCR

qRT-PCR was used to establish GM-CSF, IL-1*β*, IL-33, CXCL8, CCL2, CCL3, and TNF mRNA levels. Purified rPMCs were incubated with poly(I:C) at a final concentration of 10 *μ*g/mL or medium alone (control) for 2 h at 37°C in a humidified atmosphere with 5% CO_2_. Isolation of total RNA from rPMCs was carried out using an RNeasy Mini Kit (Qiagen, Hilden, Germany). cDNA was synthesized using High Capacity cDNA Reverse Transcription Kit TAK (Applied Biosystems, Foster City, CA, USA). For qRT-PCR, TaqMan Gene Expression Master Mix and TaqMan probes were applied. All reactions were conducted with the application of the 7900 HT Fast Real-Time PCR System (Applied Biosystems). The amplification conditions were as follows: initial denaturation at 95°C for 20 s, followed by 40 cycles of amplification: 95°C for 3 s and 60°C for 30 s. The RQ of testes samples was calculated by the SDS RQ Manager software, based on the *ΔΔ*Ct method. The expression of receptor mRNAs was corrected by normalization based on the transcript level of the housekeeping gene rat ACTB. As the calibrator samples, unstimulated specimens were used. All qRT-PCRs were performed in triplicate.

### 2.6. Histamine Release Assay

Medium-suspended purified rPMCs at a concentration of 10^5^ cells/mL were incubated with poly(I:C) at final concentrations of 0.1, 1, 10, and 100 *μ*g/mL, compound 48/80 (Sigma-Aldrich) at final concentration of 5 *μ*g/mL (positive control), or medium alone (control) for 1 h using 37°C water bath with constant stirring. After incubation, 1.9 mL of cold medium was added to stop the reaction, and the cell suspensions were centrifuged (2000 rpm, 5 min, 4°C). Once supernatants were decanted into separate tubes, 2 mL of distilled water was added to remaining cell pellets, and all samples were acidified with 3 N HCl. In another set of experiments, rPMCs were preincubated with poly(I:C) at 10 *μ*g/mL or medium alone (control) for 1 h at 37°C. Next, rPMCs were rinsed twice and treated with anti-IgE (Serotec, Oxford, UK) at a final concentration of 5 *μ*g/mL or medium alone for 30 min at 37°C. After incubation, the reaction was stopped by the addition of cold medium, and histamine content was assessed, as described above. Histamine content assessments were conducted for both cell pellets (residual histamine) and supernatants (released histamine) by the spectrofluorimetric method using *o*-phthaldialdehyde (OPT) (Sigma-Aldrich). Samples were excited at 360 nm, and fluorescence emission measured at 450 nm. Histamine release was expressed as a percentage of the total cellular content of the amine.

### 2.7. ELISA

For cytokine generation measurements, medium-suspended rPMCs (concentration: 1.5 × 10^6^ cells/mL) were incubated with poly(I:C) at final concentrations of 0.1, 1, 10, and 100 *μ*g/mL, LPS from *E. coli* (500 ng/mL), PGN from *S. aureus* (100 *μ*g/mL), or medium alone (control) for 12 h in a humidified atmosphere with 5% CO_2_. After incubation, the centrifugation (1200 rpm, 5 min, 20°C) was conducted to collect supernatants. In another series of experiments, rPMCs were challenged with anti-IgE at a final concentration of 5 *μ*g/mL, poly(I:C) (10 *μ*g/mL), both poly(I:C) and anti-IgE, or medium alone for 12 h at 37°C. Next, centrifugation was performed to collect supernatants followed by the assessment of cytokine release, as described above. Cytokine release was assessed by ELISA commercial kits for IFN-*α*, IFN-*β*, CCL3, CXCL8 (Wuhan EIAab Science Co., Ltd, Wuhan, China), or tumor necrosis factor (TNF) (Gen-Probe Inc., San Diego, CA, USA); assay sensitivities were <5.5 pg/mL, <6.1 pg/mL, <0.058 ng/mL, <7.8 pg/mL, or <15 pg/mL, respectively.

For cysLT synthesis analysis, purified rPMCs were suspended in medium to obtain concentration of 1.5 × 10^6^ cells/mL and next were incubated with poly(I:C) at final concentrations of 0.1, 1, 10, and 100 *μ*g/mL, lipopolysaccharide (LPS) from *Escherichia coli* (Sigma-Aldrich) (500 ng/mL), peptidoglycan (PGN) from *Staphylococcus aureus* (100 *μ*g/mL) (Invivogen), or medium alone (control) for 2 h at 37°C in water bath, continuously stirred. After incubation, the supernatants were collected by centrifugation (1200 rpm, 5 min, 20°C) and analyzed by ELISA commercial kit (Cayman Chemical, Ann Arbor, MI, USA) detecting cysteinyl leukotrienes (cysLTs) (LTC_4_ and its degradation products LTD_4_ and LTE_4_). In separate experiments, rPMCs were challenged with anti-IgE at a final concentration of 5 *μ*g/mL, poly(I:C) (10 *μ*g/mL), both poly(I:C) and anti-IgE, or medium alone for 2 h at 37°C. Cell suspensions were centrifuged to collect supernatants, which were then assessed for cysLT release, as described above. The concentration of cysLTs in supernatants was evaluated according to the manufacturer's instructions. The assay sensitivity was <13 pg/mL.

### 2.8. Blocking Experiments

Purified rPMCs were suspended in the medium and preincubated with goat polyclonal anti-TLR3 IgG antibodies, goat IgG isotype control antibodies (Santa Cruz Biotechnology, Santa Cruz, CA, USA) at final concentrations of 40 *μ*g/mL, nuclear factor kappa B (NF-*κ*B) inhibitor MG-132 (3 *μ*M) (Invivogen), TANK binding kinase 1 (TBK1)/I, kappa B kinase (IKK)*ε* inhibitor BX-795 (1 *μ*M) (Biomol, Plymouth Meeting, PA, USA), or medium alone (control) for 15 min (inhibitors) or 1 h (antibodies) in a humidified atmosphere with 5% CO_2_ at 37°C. Next, all samples, except those with BX-795, were washed twice before main procedure performances (i.e., cysLT and cytokine release assays).

### 2.9. Statistical Analysis

The statistical analysis of the experimental data was performed using Statistica 13 software (Statsoft Inc., USA). Data are presented as the mean ± SD. The normality of distribution was tested with the Shapiro-Wilk test. All comparisons between groups were carried out by using Student's *t*-test for small groups. Differences were considered significant at *p* < 0.05 and are labeled with an asterisk (^∗^) on each graph.

## 3. Results

### 3.1. Constitutive Expression of TLR3, IRF3, IFNAR1, IFNGR1, and MHC I Proteins in rPMCs

**Figure 1:**
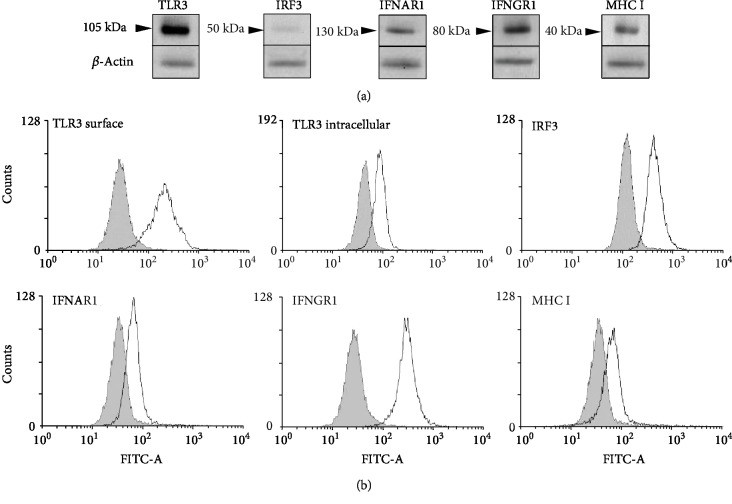
rPMCs exhibit phenotype engaged in response to viral infection. Constitutive expression of TLR3, IRF3, IFNAR1, IFNGR1, and MHC I proteins in cell lysates analyzed by Western blot. *β*-Actin was used as a loading control (a). Constitutive expression of TLR3 (surface and intracellular), IRF3, IFNAR1, IFNGR1, and MHC I molecules analyzed by flow cytometry. Shaded areas indicate staining with isotype-matched control antibodies (b). Data are representative of 3 independent experiments.

Firstly, the fully mature native MCs freshly isolated from rat peritoneal cavity were examined for constitutive expression of TLR3, IRF3, IFNAR1, IFNGR1, and MHC I proteins. Western blot analysis indicated distinct 105, 130, 80, and 40 kDa protein bands corresponding to TLR3, IFNAR1, IFNGR1, and MHC I, respectively, as well as a band at 50 kDa for IRF3 (Figure 1(a)). Flow cytometry confirmed the constitutive expression of all investigated molecules in native rPMCs (Figure 1(b)). Moreover, as we were interested in whether TLR3 protein expression occurs both intracellularly and on the cell surface, the flow cytometry assessments were conducted for permeabilized and unimpaired rPMCs. As a result, we established that the TLR3 molecule was detected in the cell interior (TLR3 intracellular) as well as in the membrane (TLR3 surface).

### 3.2. TLR3 Ligand Poly(I:C) Induces Explicit Changes in the Antiviral Response-Associated Phenotype of rPMCs

**Figure 2:**
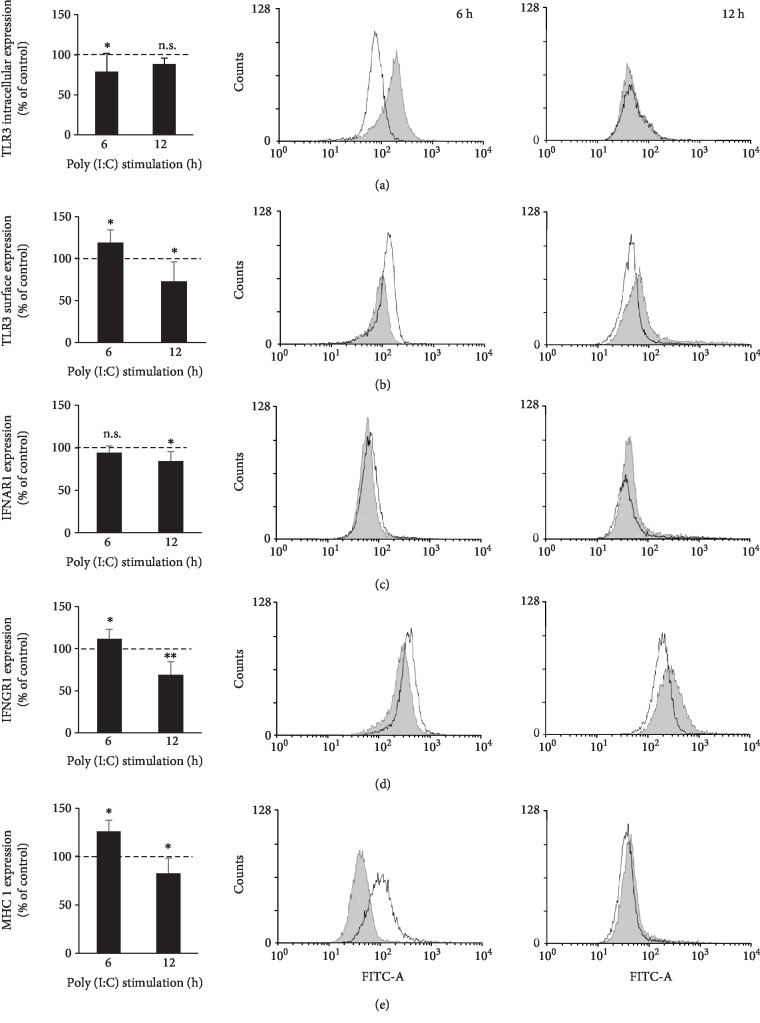
rPMCs change their phenotype upon stimulation with TLR3 ligand. rPMCs were treated with poly(I:C) at 10 *μ*g/mL or medium alone (constitutive expression) for 6 or 12 h. Expression of intracellular TLR3 (a), surface TLR3 (b), IFNAR1 (c), IFNGR1 (d), and MHC I (e) was analyzed by flow cytometry. Results are shown as percentage changes in MFI values to constitutive expression (100%). Representative flow cytometry histograms demonstrate protein expression on poly(I:C)-stimulated (white) and nonstimulated (shaded) rPMCs. Data are presented as the mean ± SD of 5 independent experiments (*n* = 5). ^∗^*p* < 0.05 and ^∗∗^*p* < 0.01; n.s.: not significant.

Next, we were interested in determining whether rPMCs respond to TLR3 ligand poly(I:C) stimulation by altering the self-expression of TLR3 (surface and intracellular), IFNAR1, IFNGR1, and MHC I protein. To this end, rPMCs were challenged with poly(I:C) (10 *μ*g/mL) for 6 h or 12 h and then analyzed by flow cytometry. As shown in Figure 2, under defined incubation times, the expression level of each protein was significantly affected upon TLR3-mediated stimulation, wherein a different nature of the resultant changes was observed. 6 h treatment of rPMCs with poly(I:C) markedly upregulated the expression of surface TLR3, IFNGR1, and MHC I, which was followed after 12 h incubation by a significant decrease, comparing to respective control (constitutive expression). Interestingly, the opposite trend was noted for the TLR3 intracellular expression level (Figure 2(a)), as it first considerably diminished (6 h dsRNA stimulation) and then almost returned to output value (12 h dsRNA stimulation). Although rPMC exposure to poly(I:C) for 6 h did not modulate IFNAR1 expression, the prolonged incubation time caused a relative decrease (*p* < 0.05). Poly(I:C) at the concentration used was not toxic for rPMCs when tested by trypan blue staining and FACS analysis at the end of the experiment (12 h).

### 3.3. rPMCs Produce Lipid Mediators, Cytokines, and Chemokines, but Do Not Degranulate, in Response to TLR3 Ligand Poly(I:C)

**Figure 3:**
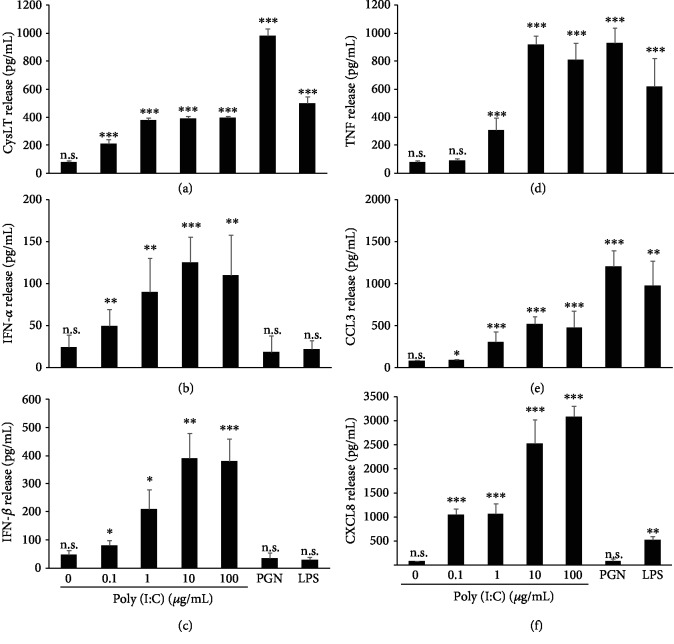
rPMCs release *de novo* synthesized mediators upon stimulation with TLR3 ligand. rPMCs were challenged with poly(I:C) at the indicated concentrations, PGN from *S. aureus* (10 *μ*g/mL), or LPS from *E. coli* (100 ng/mL) for 2 (a) or 12 h (b–f). CysLT (a), IFN-*α* (b), IFN-*β* (c), TNF (d), CCL3 (e), and CXCL8 (f) were measured in cell supernatants using ELISA. Results are presented as the mean ± SD of 4 independent experiments, and each experiment was done in duplicate (*n* = 8). ^∗^*p* < 0.05, ^∗∗^*p* < 0.01, and ^∗∗∗^*p* < 0.001; n.s.: not significant.

**Figure 4:**
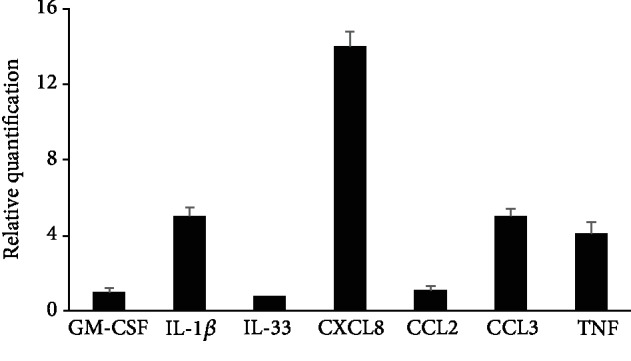
rPMCs produce a wide array of cytokine mRNAs upon stimulation with TLR3 ligand. rPMCs were challenged with poly(I:C) at 10 *μ*g/mL or medium alone (control) for 2 h. Cytokine mRNA expression was established by qRT-PCR and demonstrated as a fold increase above the value of cytokine mRNA expression in untreated cells after normalization with the transcript level of the housekeeping gene of rat Actb. Results are presented as the mean ± SD of at least 3 separate experiments performed in duplicates (*n* ≥ 6).

Since the effect of TLR3 ligation on MC biological activity remains poorly understood, we addressed the issue of whether TLR3-involved rPMCs may react *via* degranulation and preformed mediator release as well as/or *via* the synthesis of newly generated products. To evaluate degranulation, rPMCs were exposed to poly(I:C) at the concentration range (0.1-100 *μ*g/mL) for 1 h, and after that, the level of histamine release was measured. Our results indicated no significant histamine secretion at the entire range of dsRNA concentration used (data not shown). In comparison, at the same time, rPMCs challenged with compound 48/80, a potent activator of MC degranulation, released up to 56.8 ± 3.9% (mean ± SD) of total histamine content. However, we demonstrated that rPMCs synthesize and release a full range of investigated *de novo* generated mediators, i.e., cysLTs, IFN-*α*, IFN-*β*, TNF, CCL3, and CXCL8, in response to poly(I:C) in a generally dose-dependent manner (Figure 3). Even dsRNA concentration as low as 0.1 *μ*g/mL activated rPMCs to secretes a significant amount of cysLTs, IFN-*α*, IFN-*β*, CCL3, and CXCL8, whereas TNF release was observed only at ≥1 *μ*g/mL of poly(I:C) concentration. Notably, the highest TLR3-mediated synthesis was observed for CXCL8 (up to 2926.1 ± 422.9 pg/mL) at the poly(I:C) concentration equal to 100 *μ*g/mL (Figure 3(f)). To investigate the selectivity of TLR-mediated secretion, the cells were additionally treated with PGN (TLR2 ligand) and LPS (TLR4 ligand). As expected, both ligands induced cysLTs, TNF and CCL3, but did not affect IFN-*α* and IFN-*β* secretion. None or low CXCL8 release upon rPMC treatment with PGN or LPS, respectively, was also stated. Induction of some proinflammatory cytokines and chemokines in dsRNA-stimulated rPMCs was confirmed at the mRNA level, as well. For those studies, rPMCs were stimulated with poly(I:C) at 10 *μ*g/mL for 2 h, and total RNA samples were analyzed using qRT-PCR. As demonstrated in Figure 4, dsRNA strongly induced interleukin- (IL-) 1*β*, TNF, CCL3, and CXCL8 mRNA expression in rPMCs, which stayed in line with the results obtained for the secretion assessments.

### 3.4. Involvement of TLR3, NF-*κ*B, and IRF3 in TLR3 Ligand Poly(I:C)-Induced Mediator Synthesis in rPMCs

**Figure 5:**
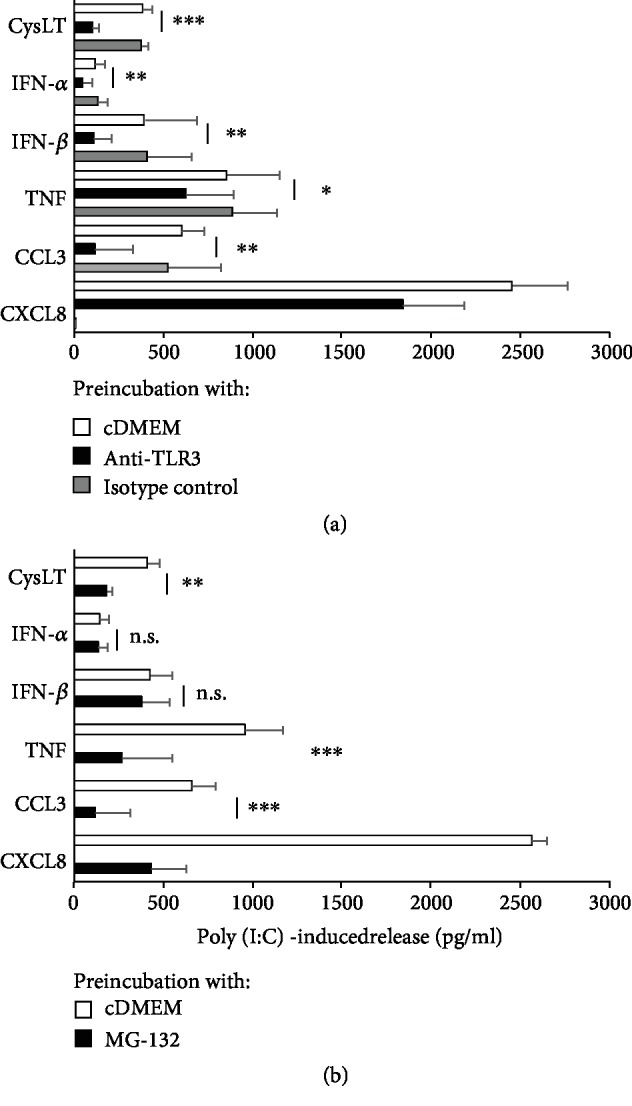
TLR3 ligand-induced release of *de novo* synthesized mediators from rPMCs is TLR3- and NF-*κ*B-dependent. rPMCs were preincubated with medium alone (control), anti-TLR3 antibodies (40 *μ*g/mL), or isotype control antibodies for 1 h and after rinsing exposed to poly(I:C) at 10 *μ*g/mL for 2 h (cysLT release) or 12 h (cytokine release) (a). rPMCs were preincubated with medium alone (control) or MG-132 (3 *μ*M) for 15 min and after rinsing treated with poly(I:C) at 10 *μ*g/mL for 2 h (cysLT release) or 12 h (cytokine release) (b). Results are reduced by the value of the spontaneous release. Data are expressed as the mean ± SD of 4 independent experiments, and each experiment was done in duplicate (*n* = 8). ^∗^*p* < 0.05, ^∗∗^*p* < 0.01, and ^∗∗∗^*p* < 0.001; n.s.: not significant.

**Figure 6:**
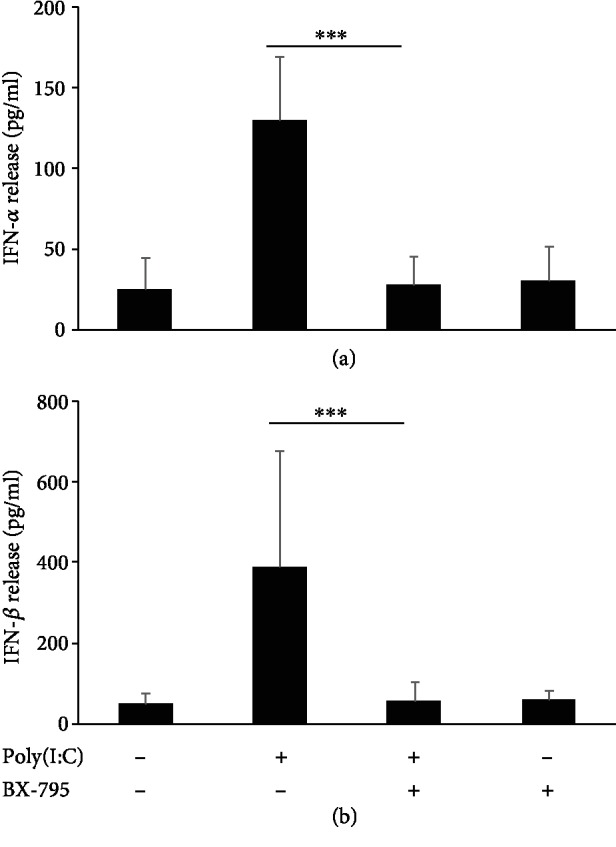
TLR3 ligand-induced release of type I IFNs from rPMCs is TBK1/IKK*ε*-dependent. rPMCs were preincubated with medium alone (control) or BX-795 (1 *μ*M) for 15 min and then exposed to poly(I:C) at 10 *μ*g/mL for 12 h. IFN-*α* (a) and IFN-*β* (B) concentration was measured in cell supernatants using ELISA. Data are presented as the mean ± SD of 4 independent experiments, and each experiment was done in duplicate (*n* = 8). ^∗∗∗^*p* < 0.001.

TLR3 stimulation may activate downstream transcription factors IRF3 and NF-*κ*B *via* IKK*ε*/TBK1 and IKK*α*/*β*, respectively [37]. To establish the specificity of the dsRNA-induced effect on mediator generation in rPMCs, we conducted series of blocking experiments using anti-TLR3 blocking antibodies and appropriate inhibitors (MG-132 for NF-*κ*B activation and BX-795 for IKK*ε*/TBK1 activation). We established that the release of all examined mediators in response to poly(I:C) (10 *μ*g/mL) was significantly and differentially decreased by TLR3 blocking (Figure 5(a)). Similarly, rPMC pretreatment with MG-132 sharply reduced the dsRNA-induced level of cysLTs, TNF, CCL3, and CXCL8 but, as expected, did not affect IFN-*α* and IFN-*β* secretion (Figure 5(b)), which in turn was wholly inhibited by BX-795 (Figure 6). Importantly, DMSO-diluted BX-795 showed no effect on direct IFN-*α* and IFN-*β* release and was not toxic for rPMCs when checked by trypan blue staining.

### 3.5. TLR3 Ligand Poly(I:C) Modulates Fc*ε*RI-Dependent cysLTs and TNF Synthesis from rPMCs

**Figure 7:**
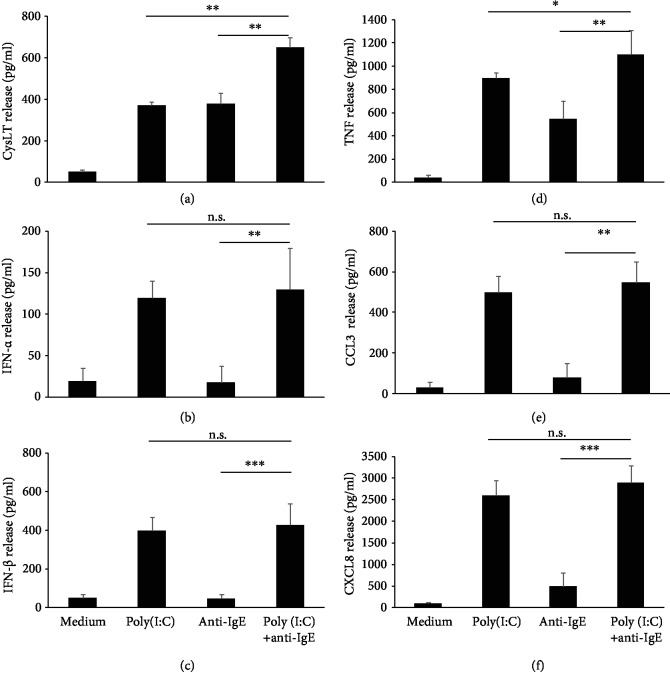
Fc*ε*RI-mediated release of *de novo* synthesized mediators upon rPMC stimulation with TLR3 ligand. rPMCs were treated with medium alone, poly(I:C) at 10 *μ*g/mL, anti-IgE at 5 *μ*g/mL, or both poly (I:C) and anti-IgE for 2 (a) or 12 h (b–f). CysLT (a), IFN-*α* (b), IFN-*β* (c), TNF (d), CCL3 (e), and CXCL8 (f) were measured in cell supernatants using ELISA. Bars for the poly(I:C) at 10 *μ*g/mL and medium alone demonstrate the same data set as in Figure 3. Results are presented as the mean ± SD of 4 independent experiments, and each experiment was done in duplicate (*n* = 8). ^∗^*p* < 0.05, ^∗∗^*p* < 0.01, and ^∗∗∗^*p* < 0.001; n.s.: not significant.

It has long been documented that viral infections exacerbate the course of allergic processes, including bronchial asthma [38, 39], but the underlying cause of this phenomenon remains to be identified. Therefore, it might be speculated that TLR3-specific viral ligands are capable of modulating Fc*ε*RI-mediated MC response. To gain further insights into the issue, we examined the influence of poly(I:C) on the Fc*ε*RI-dependent release of preformed and *de novo* synthesized mediators from rPMCs. As a result, we established that 1 h priming of native rPMCs with poly(I:C) at 10 *μ*g/mL did not affect anti-IgE-induced rPMC degranulation (data not shown). By contrast, we found that costimulation of rPMCs with TLR3 agonist and anti-IgE considerably amplified cysLTs and TNF but did not affect IFN-*α*, IFN-*β*, CCL3, and CXCL8 secretion, as presented in Figure 7.

## 4. Discussion

There is a wealth of data identifying MCs as a crucial player in the host innate and adaptive immune response to bacterial infection [4, 7, 26, 40]. Incomparably less is known about the role of those cells in the mechanisms of antiviral immunity or/and the pathomechanism of viral-related diseases. Certainly, MCs can directly respond to emerging viruses. They are strategically placed at the junction point of the host and external environment; therefore, they belong to the first immune cells, which encounter pathogens, therein viruses [1]. MCs recognize the viral products through the multiplicity of cell surface/intracellular receptors, i.e., TLR3, TLR7, TLR9, and RIG-I-like molecule, which have the capacity to the detection of virus-derived molecular patterns [9]. TLR3 mainly responds to dsRNA from the viral genome presented extracellularly; TLR7 and TLR9 recognize viral ssRNA and unmethylated CpG motifs within DNA, respectively. The latest described PRR receptor in MCs is RIG-I which senses short (<300 bp) 5′-triphosphorylated dsRNA or ssRNA with double-stranded regions. The leading contribution of MCs in response to viral infection might be in the context of the producing panel of mediators (amines, tryptase, chymase) and cytokines/chemokines (TNF-*α*, IL-4, IL-5, IL-6, IL-13, and IL-17) [2, 4]. Those cells are an essential and indisputable local source of IFNs (type I and III) following viral challenge. Beyond direct antiviral effects, MCs can detect infecting viruses indirectly by sensing danger signals released from infected cells (alarmins) and mediators produced in the context of the antiviral response. Since MCs are an abundant source of diverse biologically active mediators, such as granule-associated preformed mediators, *de novo* generated arachidonic acid metabolites, and many newly synthesized cytokines and chemokines, they can alter the functions of the surrounding cells and tissues. MC-derived proinflammatory mediators, cytokines, and chemokines elicit the development of inflammation at the site of pathogen entry [4]. MCs promote the antiviral host defense by enrolment and supporting additional effector immune cells, e.g., natural killer (NK) cells, NKT cells, and CD8^+^ T cells. Mechanisms of MC antiviral response have been studied in different experimental infection models in mouse and human cell culture systems [7]. MCs have been reported to be targets of dengue, influenza A, human *immunodeficiency*, and hepatitis viruses resulting in degranulation and robust cytokine and chemokine response. Interestingly, MC activation by cytomegalovirus (CMV) occurs in two sequences of degranulation: a sharp early MC degranulation requiring TLR3/TRIF (TIR domain-containing adapter-inducing interferon-*β*) and later TLR3/TRIF-independent degranulation, most likely in reaction to viral replication [41]. Additionally, MCs act as players in a cross-talk axis between innate and adaptive immune control of CMV [42]. This prompted us to examine MCs for attributes as well as for various aspects of their activity that could have potential significance in the processes developed during viral infection.

In MCs, TLR3 expression was yet demonstrated mainly at the transcript level [27–29, 31, 43], whereas only a few reports described the protein [27, 31, 44]. To our knowledge, the exclusive study indicating TLR3 molecule, both intracellular and surface, in native mature murine MCs was suggested by Orinska et al. [31]. Previously, we reported the mRNA and protein TLR3 expression in *in vivo* differentiated mature tissue MCs isolated from the rat peritoneal cavity [33]. In our research, by the use of western blot and flow cytometry techniques, we have confirmed that fully mature native rPMCs express TLR3 protein, wherein by the second method, we additionally confirmed the presence of TLR3 molecule both intracellularly and on the cell surface. All these data indicate that MCs may recognize and bind viral dsRNA. Furthermore, we found that the TLR3 ligation with dsRNA synthetic mimic, i.e., poly(I:C), after 6 h caused the increase in the surface TLR3 expression while at the same time reduced its intracellular level. Interestingly, it was observed that the activity ceased within the next 6 h of stimulation. This temporary inversion relationship might be explained by the process, in which some portion of intracellular TLR3 is translocated toward the cell membrane, thereby supplying its surface level. A poly(I:C)-induced increase in the expression of accessory protein UNC93B1, known for its function in translocation of nucleic acid-sensing (NAS) TLRs to endosomes [45], was implicated in selective TLR3 trafficking to plasma membrane resulting in the upregulation of surface TLR3 expression in human umbilical vein endothelial cells (HUVEC) [46]. Such translocation could be the underlying cause in the previously observed increase of TLR3 surface expression for airway epithelial cells (A549) exposed to the respiratory syncytial virus [47] and for human bronchial epithelial cells (BFAS-2B) infected with rhinovirus [48]. Importantly, poly(I:C)-activated upregulation of TLR3 expression was also demonstrated in murine bone marrow-derived cultured MCs (BMMCs), though only at the mRNA level [31]. Furthermore, poly(I:C) stimulated a six-fold increase in the total number of lung MCs *via* TLR3 [49].

Our findings also indicated the presence of MHC I molecule on the rPMC surface, similar to BMMCs, as previously shown [23]. Given the hitherto evidence for MC's ability to present antigens *via* MHC I *in vitro* [24] and *in vivo* [23], these cells might be suspected to promote the development of the adaptive response to viruses by presenting endogenous antigens of viral origin to CD8^+^ T lymphocytes. Furthermore, our results for the temporary increase in surface expression of MHC I in poly(I:C)-exposed rPMCs further imply that dsRNA recognition by MCs could temporarily enhance the capacity of viral antigen presentation. Interestingly, observed changes in MC phenotype after 6 h stimulation with dsRNA were followed by a sharp decline after 12 h, even below the control constitutive expression level. Thus, we may venture the hypothesis that rPMCs have autoregulation mechanisms, which after the response period, effectively silence dsRNA-induced phenotype, making these cells less reactive in the further course of antiviral response.

What appears to be particularly intriguing is whether TLR3 in MCs serves as a functionally active receptor capable of mediating cell activation to generate and release various mediators that promote inflammation or/and modulate the activity of other immune cells. We established that poly(I:C) did not induce rPMCs to degranulation and, consequently, to the release of preformed proinflammatory products, which is in line with other studies [27, 28, 31, 43]. On the other hand, we found that these cells secrete a relatively small amount of highly proinflammatory cysLTs. It should be noted, however, that these lipid mediators may provoke pronounced physiological effects even at nanomolar concentrations [50]. As far as we know, heretofore, only the report by Kulka et al. [27] has documented cysLT generation by MCs, though it was not confirmed in the later studies [43, 51]. Murine fetal skin-derived cultured MCs (FSMCs) release CCL3, CCL4, CCL5, IL-6, and TNF-*α* upon TLR3-poly(I:C) activation [10, 28]. Peritoneal MCs from C57BL/6 mice activated by poly(I:C)-TLR3 have increased CCL5 and CXCL10 expression [31]. In this paper, we also demonstrated that poly(I:C)-challenged rPMCs synthesize *de novo* proinflammatory cytokines and chemokines, i.e., IL-1*β*, TNF, CCL3, and a particularly high amount of CXCL8. Noteworthy, we stated significant differences in the secretion level of investigated mediators when these cells were challenged with TLR2 and TLR4 ligands, i.e., PGN and LPS, respectively, which underlines TLR functional distinction. Moreover, in observed cytokine and chemokine, and also cysLTs, the release was significantly inhibited upon anti-TLR3 blocking antibodies or NF-*κ*B inhibitor providing evidence for the specificity of poly(I:C)-induced rPMC response. To date, murine and human MCs were shown to produce and secrete TNF, IL-6, and IL-13 [28, 44] as well as several chemokines, including CCL2, CCL3, CCL4, CCL5, CXCL1, CXCL2, CXCL8, and CXCL10 [28, 31, 43, 44, 51], though in some cases data are conflicting. Furthermore, present reports are in agreement with the lack of IL-1*β*, IL-5, granulocyte-macrophage colony-stimulating factor (GM-CSF), IL-29, CXCL9, and CXCL11 induction in TLR3-activated MCs [27, 51, 52]. This allows us to believe that MC stimulation *via* TLR3 leads to the synthesis of multiple potent mediators entailing the development of inflammation, which is a crucial defensive mechanism during viral infections. Fundamental importance in the process may have secreted in a large amount by dsRNA-stimulated MCs CXCL8, which was strongly evidenced for the induction of CD8^+^ T and NK cell influx leading to viral clearance [31, 51].

One of the major components of cellular response to virus infection is IFNs, as their primary role is to restrict virus replication within the infected cells and induce an antiviral state in uninfected cells. Regarding MCs, the capability to produce IFNs still raises some doubts [53, 54]; therefore, we were primarily interested in whether dsRNA analog induces type I IFN production and release from rPMCs. Although previously TLR3-mediated secretion of IFN-*α* and IFN-*β* was demonstrated for human and murine MCs [27], this single finding was not corroborated in later studies [54]. Herein, we clearly showed that rPMCs release IFN-*α* and IFN-*β* in response to poly(I:C) stimulation as well as express IRF3 responsible for IFN gene expression. Although IFN secretion was TLR3-dependent as with other examined dsRNA-induced mediators, the signal transduction occurred through IKK*ε*/TBK1, but not NF-*κ*B, which means the specific engagement of distinct IRF3-associated pathway.

Furthermore, type I IFN synthesis was not induced in rPMCs treated with TLR2 and TLR4 ligand, indicating its restriction to TLR3 stimulation. The lack of kind I IFN synthesis in MC response to LPS stimulation was suggested by others, as well [53, 54]. In this report, we also prove that MCs not only produce IFNs but also possess relevant surface receptors to receive their signal, i.e., IFNAR for type I IFNs and IFNGR for type II IFN. Although there is little data on IFN receptor expression by MCs, there is strong evidence for the direct effect of IFN type I and II on these cells [55–57]. Our findings for the upregulation of IFNGR1 expression in poly(I:C)-treated rPMCs may confirm that these cells raise their sensitivity to IFN-*γ* message when exposed to dsRNA-type virus infection. Collectively, we may point out that MCs produce type I IFNs and, at the same time, are activated by type I and II IFNs derived from other cell populations, thereby making their contribution to the extensive cellular network of antiviral immunity.

What is intriguing is that the TRIF- and MyD88-dependent signaling branches in the TLR3 and TLR9 pathway can collaborate to induce an effective innate immune response. Tabeta et al. [58] observed that the weakness of either TLR3 or TLR9 signaling pathways has an immense effect on the course of the CMV infection in HEK 293 cells. Impairment of TLR3 signaling causes a >60% reduction, and abrogation of TLR9 signaling causes a >90% decrement in the amount of IFN (type I); hence, the two pathways seem to induce the production of type I IFN in a superadditive or codependent manner. On the one hand, it would seem that either pathway would replenish the loss of the other; however, it may be that both pathways are essential for containment of infection. Whitmore et al. [59] have made similar observations. In support of IFN-independent mechanisms regulating synergy, cotransfection of human TLR3 and TLR9 caused a synergistic activation of the *IL-8* reporter construct in response to dsRNA stimulation in murine macrophages, suggesting that components of TLR3 and TLR9 signaling pathways can combine synergistically. It will be of interest to determine whether synergism occurs through mechanisms such as TLR3/TLR9 heterodimerization and corecruitment of the TLR adapter molecules TRIF for TLR3 and MyD88 for TLR9 in MCs.

It is a commonly held belief that viral infections induce exacerbation of bronchial asthma [38, 39], but the underlying cause is yet unknown. In this regard, we examined the effect of TLR3 ligation on Fc*ε*RI-dependent mediator synthesis and/or release by rPMCs. We documented that poly(I:C) priming had no impact on anti-IgE-induced degranulation in these cells. On the other hand, rPMC simultaneous treatment with TLR3 agonist and anti-IgE amplified cysLT and TNF secretion. Previous studies indicated that poly(I:C) pretreatment did not affect IgE-dependent degranulation of human peripheral blood-derived cultured MCs (HCMCs) and LAD cells, but slightly augmented antigen-dependent secretion of cysLTs, TNF, IL-1*β*, and IL-5 from HCMCs [27]. However, the report by Becker et al. [41] indicated that only prolonged, up to 96 h, exposure to poly(I:C) induced a significant increase in Fc*ε*RI-mediated release of *β*-hexosaminidase as well as generation of cysLTs and LTB_4_ by connective tissue-like MCs (CTLMCs) and mucosal-like MCs (MLMCs). The observation that IgE-activated cysLTs and TNF release is elevated upon MC response to viral dsRNA analog might be of great importance due to the known pathobiological role of these mediators in severe asthma [60]. Pathogen-derived components and allergens are significant inducing factors of allergic disorders; not surprisingly, then, the potential existence of cross-talk between TLR and Fc*ε*RI signaling pathways is widely speculated [10, 61]. The ultimate cell response is determined by the processing of the intracellular signal network, which arises from the stimulation of many receptors on the same cell. Thus, MC parallel stimulation of TLR and Fc*ε*RI could induce a synergistic effect on the release of proinflammatory mediators. Thesis on augmented IgE-dependent releasability due to increased Fc*ε*RI expression upon dsRNA stimulation remains somewhat questionable. As it was shown by Kulka and Metcalfe [29], poly(I:C) stimulation did not induce the upregulation of Fc*ε*RI expression on human MC line LAD1.

## 5. Conclusion

To conclude, we demonstrated that rPMCs express intracellular and surface TLR3 binding viral dsRNA as well as possess other molecules associated with cellular antiviral response. Furthermore, rPMC was shown to respond through TLR3 by altering their phenotype and synthesizing proinflammatory mediators, cytokines, and chemokines as well as, most importantly, by secreting type I IFNs. We also proved that rPMCs are capable of being stimulated by IFNs as the relevant type I and II IFN receptors were indicated on their surface. Finally, data presenting that Fc*ε*RI-mediated releasability of rPMCs might be modulated upon TLR3 ligation imply that dsRNA-type viruses may influence the severity of allergic reactions. These findings bring us closer to regarding MCs as active participants in the antiviral immune response, but also, we should bear in mind their negative role in the pathological events during viral infections. Nonetheless, the current data on this issue is ambiguous and partial; thus, further studies are required.

## Data Availability

The data used to support the findings of this study are included within the article.
